# Assessment of mandatory declaration excipients in pediatric off label prescriptions in Spain

**DOI:** 10.1038/s41598-025-11647-x

**Published:** 2025-07-19

**Authors:** Irene Lizano-Díez, Itziar Aldalur-Uranga, Antonio J. Braza, Carlos Figueiredo-Escribá, Montserrat Viñas-Bastart, Eduardo L. Mariño, Pilar Modamio

**Affiliations:** 1 Clinical Pharmacy and Pharmaceutical Care Unit, Department of Pharmacy and Pharmaceutical Technology, and Physical Chemistry, Faculty of Pharmacy and Food Sciences, University of Barcelona, Av. Joan XXIII, 27-31, Barcelona, 08028 Spain; 2 Clinical Pharmacy and Pharmacotherapy (FCFT) Research Group 2021 SGR 01082, Faculty of Pharmacy and Food Sciences. University of Barcelona, Barcelona, Spain

**Keywords:** Pediatrics, Safety, Excipient of mandatory declaration, Primary care, Off-label prescription, Paediatric research, Drug therapy

## Abstract

Pediatric patients are at increased risk of exposure to potentially harmful excipients when receiving off-label medicinal products primarily formulated for adults, raising concerns about adverse events. Based on data from a previously published study, this research focuses on the most frequently prescribed off-label medicinal products in order to assess the safety of their excipients. The aim was to identify Excipients of Mandatory Declaration (EMDs) that could pose additional safety risks in individuals under the age of 18. A descriptive, and retrospective observational population-based study in individuals under 18 was developed in the Spanish primary care setting between the years 2004–2005 and 2017–2020. The main data source was proprietary database, with nationwide coverage of prescriptions. The analysis included 4,922,984 off-label prescriptions, accounting for 16 active ingredients and 37 medicinal products (18 oral, 16 topical and three inhaled) that were scrutinized for EMD composition. Neonates and infants up to five years old accounted for nearly 50% of all off-label prescriptions among the under-18 population, exposing them to additional safety risks from potentially harmful EMDs in these formulations, which may lead to severe adverse events, causing permanent or long-term harm. The degree of harm of EMDs was assessed overall as “severe” for nine out of 22 EMDs, mostly used for oral formulations, and “moderate” for six EMDs. No EMDs were categorized as “no harm”. Results on EMDs in oral medicinal products, for instance, highlight the urgent unmet needs in this regard, especially for young children; EMDs like sodium, sorbitol, propylene glycol and sweeteners often exceeded EMA-recommended thresholds. This study underscores the need to address risks linked to off-label use of medicinal products in children, particularly those related to EMDs. Integrating excipient risk warnings into prescribing systems and monitoring related adverse events is strongly recommended.

**Supplementary Information:**

The online version contains supplementary material available at 10.1038/s41598-025-11647-x.

## Introduction

Excipients are inert substances that are mixed with the active ingredient(s) to make up the medicinal product, contributing to their consistency, shape, flavour, and other features that facilitate manufacturing and storage^1,2^. While generally regarded as inert” and lacking therapeutic effects, some excipients can have recognized actions that may lead to adverse events, particularly in individuals with specific allergies or intolerances. To address this, the European Medicines Agency (EMA) has established a list of “Excipients of Mandatory Declaration” (EMDs) that must be specified in the Summary of Product Characteristics (SmPC), Package Leaflet (PL), and on labelling, highlighting the risk of unintentional ingestion^3,4^. Similarly, the Spanish Agency of Medicines and Medical Devices (AEMPS), as a member state of the European Union (EU), adheres to this mandate^5^.

It has been almost 18 years since the publication of the Pediatric Regulation (1901/2006/EC) in Europe. The objective of this regulation was to ensure that adequate studies are carried out in neonates, children, and adolescents up to 17 years of age to obtain the necessary data for the assessment of risks and benefits for the authorization of medicinal products for the under-18 population^6^.

On a previous publication, Lizano-Díez and cols^7^. conducted an observational study within the primary health care context to analyse annual prescription rates among the under-18 population in Spain. The study compared four cross-sectional annual periods: one prior to the implementation of the Pediatric Regulation (October 2004 – September 2005) and three after (October 2017-September 2020) to allow sufficient time to observe potential changes. The authors scrutinized off-label prescriptions according to the age of use that was outlined in the SmPC and sought to identify changes in off-label prescription rates in Spain following the Regulation’s implementation; however, they did not observe any meaningful decrease. In addition to the challenges posed by off-label prescriptions, the under-18 population may be exposed to harmful excipients present in formulations primarily designed for adults, potentially leading to additional adverse events. Therefore, building on the findings from the research of Lizano-Díez and cols^7^., it was sought relevant to analyse the excipients from the most prescribed off-label medicinal products and identify which EMDs may represent a potential additional safety risk for individuals under 18.

## Method

### Study design and setting

A descriptive, and retrospective observational population-based study was developed by analysing the EMDs in medicinal products prescribed off-label to the population under 18 years of age in the Spanish primary care setting identified in a pre-post study^7^ between the years 2004–2005 and 2017–2020.

### Inclusion criteria

Prescriptions of off-label medicinal products issued by pediatricians to Spanish newborns, children and adolescents up to 17 years of age who were treated as outpatients in primary care setting. No limits were established on patient’s sex or location within the Spanish geography.

Only off-label prescriptions related to branded medicinal products, either innovative or generic, were considered for the EMD analysis.

### Exclusion criteria

Any prescriptions of off-label medicinal products issued by health professionals other than pediatricians, such as general practitioners (GPs) and nurses. Prescriptions for inpatients were also excluded from the analysis. Only branded medicines were included, while prescriptions issued by active ingredient name (e.g., unbranded ibuprofen) were excluded, as identifying their excipients was not feasible given the wide variety of available generic and innovator versions.

### Data sources

As commented before, the data collection of the study incorporated raw data from the previous analysis from Lizano-Díez and cols^7^. published in April 2021, which identified off-label medicinal products prescribed in the pediatric population in Spain. The objective was two-fold: (1) to retrieve data from a one-year period preceding the implementation of the Pediatric Regulation in Europe (2006); selecting October 2004 to September 2005 as an appropriate reference; and (2) to collect data from several annual periods following the regulation’s implementation—specifically October 2017 to September 2020—to allow sufficient time to observe potential changes and because it represented the most recent data available when preparing the manuscript for submission. The authors depicted the top 10 most prescribed active ingredients per study period (*n* = 4), which resulted in a blended total of 16 individual active ingredients (i.e., acetylcysteine, benzydamine, budesonide, ciclopirox, colecalciferol, dexamethasone and anti-infectives, dexchlorpheniramine, domperidone, ketoconazole, ibuprofen, mepyramine theophyllinacetate, methylprednisolone aceponate, omeprazole, paracetamol, silicones, terbinafine).

Study data collection included the units dispensed under medical prescription to evaluate the prescribing tendency from a proprietary database, with nationwide coverage of prescriptions. sorted by patient’s age and medicinal product (branded name, active ingredient, strength, package and dosage form) and was not linked to medical records.

The SmPC was the primary source to identify the EMDs in the off-label medicinal products prescribed, or the PL if not available in the Medicine Online Information Center (CIMA) of the AEMPS^8^. It is an application that lets include queries under different criteria, so that different levels of detail of medicinal products information are obtained, like composition.

The Anatomical Therapeutic Chemical Classification System (ATC) codes and dispensation requirements for each medicinal product were also retrieved from the CIMA of AEMPS^8^.

An additional data source that was consulted for consistency and quality check of the information was BotPlus 2.0, the catalogue of the General Council of Official Colleges of Pharmacists (CGCOF)^9^. Likewise, up-to-date European regulation^4^ and scientific articles that dealt with the topic of EMDs were also consulted to collect more information on the topic.

### Variables

For each medicinal product prescribed off-label in the pediatric population, the following qualitative variables were collected: ATC code (i.e., pharmacological subgroup, ATC third level; chemical subgroup, ATC forth level; active ingredient, ATC fifth level), strength, package, and dosage form, EMDs and purpose of the excipient in the formulation.

The ATC classification is included as a descriptive variable, offering relevant insights into the route of administration— which underpins the structure of the results and discussion sections — as well as the therapeutic indications for off-label use of the medicinal products. This approach also facilitates comprehension for new readers regarding the therapeutic areas under investigation and ensures consistency with the discussion presented in the previous pre-post study of reference^7^.

The quantitative variables collected were the following: number of total prescriptions, % of prescriptions over total, maximum daily dose of the EMDs (threshold), amount of EMD per dosage unit.

### Data analysis

A descriptive statistical analysis of the data was carried out. Quantitative variables were published as totals and percentages. Percentages were calculated for characterization of medicinal products prescribed, and each formula is indicated in table’s footnotes.

The information was then cross-referenced with European regulation^4^ and several studies on the suitability of the EMDs used in pediatrics to determine the additional risk for this population group. Adapting the criteria from the National Reporting and Learning System (NRLS) from the United Kingdom (UK), a voluntary reporting system for patient safety incidents, EMDs were classified according to the degree of harm for pediatric individuals into four categories, being: (a) “no harm”, (b) “low” (minimal harm, patients may require extra observation in case of local reactions or mild allergies), (c) “moderate” (short-term harm, patients may require further treatment or procedure), and (d) “severe” (permanent or long-term harm)^10^.

Finally, based on either the SmPC or PL for each of the medicinal products, the average daily intake was either retrieved (e.g., from SmPC and/or PL)^8^ or calculated, and compared with the maximum daily doses on EMDs of the medicinal products.

### Ethical aspects

According to Spanish regulation (Royal Decree 957/2020, of November 3, regulating observational studies with medicinal products for human use), studies related to the units dispensed under medical prescription to evaluate the prescribing tendency are not legally required to obtain permission from the Ethical Review Committee or register the study protocol. Informed consent was also not necessary to request from subjects and/or their legal guardian(s) since an Ethical Review Committee approval of a protocol was not required at this study. All methods were carried out in accordance with relevant guidelines and regulations.

## Results

### General results

Out of the total 7,882,227 off-label prescriptions depicted by Lizano-Díez and cols^2^. about the most prescribed active ingredients in the four annual periods studied (*n* = 16), there were 2,959,243 prescriptions where it was not possible to assess EMDs due to their nature of prescription by active ingredient (37.5% of total off-label prescriptions).

From the remaining 4,922,984 off-label prescriptions analysed, 22 different EMDs were identified from 37 medicinal products that were prescribed off-label (18 oral, 16 topical (e.g., 13 cutaneous, one nasal, one eye, one vaginal) and three inhaled). All of them were branded innovator medicinal products except one branded generic (e.g., calcium + vitamin D 400 IU/1,500 mg chewable tablets). Each active ingredient was included in different medicinal products (i.e., such as different dosage forms, routes of administration, brand names), hence the 16 active ingredients resulted in a total of 37 different medicinal products.

Of the 37 off-label medicinal products analysed, 14 were prescribed consistently across all four study periods, and two appeared in three of the periods, including 2004–2005. Only four products were prescribed exclusively during the 2004–2005 period; however, two of these were excluded due to the absence of EMDs, making the overall impact of off-label products prescribed solely in the first period minimal. Notably, all medicinal products from 2004 to 2005 remain commercially available today.

The detail on the activity of each EMD is disclosed in Supplementary Material 1. The degree of harm of EMDs was assessed as “severe” for nine of them (e.g., aspartame, benzoates, ethanol, glucose, lactose, propylene glycol and their esters, sodium, sorbitol and sucrose) when used for oral formulations, and “moderate” for six EMDs (e.g., azo colouring agents, benzalkonium chloride, benzyl alcohol, parahydroxybenzoates and their esters, polysorbates and sulphites including metabisulphites). No EMDs were categorized as “no harm” for the pediatric study population^4,11,12^. The results section was significantly shaped by a particular emphasis on patients under five years of age.

Out of the 37 medicinal products analysed, nine did not contain in their composition any EMD, accounting for 3,186,300 prescriptions (almost 65% of total off-label prescriptions analysed) (Table 1). It is worth noting that ciclopirox, methylprednisolone aceponate, and paracetamol were also present in other medicinal products containing EMDs.

**Table 1:** Off-label medicinal products not including EMDs in their composition.

Medicinal product	Number of prescriptions (% of total) *	Route of administration	ATC code
Paracetamol 500 mg tablets	268,590 (5.5%)	Oral	N02B - Other analgesics and antipyretics n02be - anilides n02be01 - paracetamol
Methylprednisolone aceponate 0.1% solution**	17,676 (< 1%)	Topical (cutaneous)	d07a - corticosteroids, monopharmaceuticals D07AC – Potent corticosteroids (group III) D07AC14 - Methylprednisolone aceponate
Methylprednisolone aceponate 0.1% ointment	12,530 (< 1%)	Topical (cutaneous)	
Methylprednisolone aceponate 0.1% solution**	26,824 (< 1%)	Topical (cutaneous)	
Benzydamine 500 mg powder for vaginal solution	49,359 (1%)	Topical (vaginal)	G02C - Other gynecological products G02CC - Anti-inflammatory products for vaginal administration G02CC03 - Benzydamine
Cholecalciferol 25,000 IU solution	1,670,993 (34%)	Oral	A11C - Vitamins A and D, including combinations of the two A11CC - Vitamin D and analogues A11CC05 - Cholecalciferol
Mepyramine theophyllineacetate 150 mg capsules	939,577 (19%)	Oral	R03D - Other agents against obstructive diseases of the digestive system R03DA - Xanthines R03DA12 - Mepyramine theophyllineacetate
Dexchlorpheniramine 5 mg/mL solution	199,125 (4%)	Oral	R06A - Antihistamines for systemic use R06AB - Substituted alkylamines R06AB02 - Dexchlorpheniramine
Ciclopirox 80 mg/g medicated nail lacquer	1,626 (< 1%)	Topical (nail)	D01A - Antifungals for topical use D01AE - Other antifungal preparations for topical use D01AE14 - Ciclopirox

Notes: *Considering total off-label prescriptions analysed = 4,922,984. Calculation of % of total = [Number of prescriptions by medicinal product/Total number of off-label prescriptions] *100; **Refers to two different brands with same composition and indications | Abbreviations: ATC: Anatomical Therapeutic Chemical Classification System; g: Gram; IU: International Units; mg: Milligram; mL: Millilitre; SmPC: Summary of Product Characteristics | Sources^8^:.

Out of the 1,736,684 off-label prescriptions analysed with potentially harmful EMDs, nearly 50% were issued for patients under five years of age. Furthermore, more than 30% of these off-label prescriptions were for infants younger than three years.

### Oral medicinal products

Out of the 18 oral medicinal products which were prescribed off-label, most of them (*n* = 10) were tablets (either oral disintegrating tablets, effervescent tablets, chewable tablets or conventional tablets). As previously depicted in Table 1, there were four oral medicinal products that were prescribed off-label but did not contain any EMD. More details on the 14 oral off-label medicinal products analysed (*n* = 566,629 prescriptions; 11.5% of total off-label prescriptions analysed) are available in Supplementary Material 2.

The highest rate (equal or higher than 50%) of those off-label prescriptions in children under five years of age corresponded to simethicone solution (89%), followed by domperidone suspension (69%) and calcium + vitamin D chewable tablets (66%), which in turn included in their composition *five or more* different EMDs each.

### Topical medicinal products

Out of the 16 medicinal products for topical use which were prescribed off-label, five did not contain any EMD in their composition; the 11 remaining were nine medicinal products for cutaneous use (e.g., three creams, three medicated shampoos, one emulsion, one solution and one gel) and two medicinal products for non-cutaneous use (one eye drops and one nasal spray). More details on total off-label medicinal products analysed for cutaneous use (*n* = 478,432 prescriptions; 9.7% of total off-label prescriptions analysed) are available in Supplementary Material 3.

The highest rate (equal or higher than 50%) of those off-label prescriptions in children under five years of age corresponded to methylprednisolone aceponate, either emulsion or cream (54%), followed by ketoconazole, either cream or gel (51%).

Table 2 shows the topical but non-cutaneous medicinal products with EMDs in their composition. This category was represented by two medicinal products, accounting for a total of 364,827 off-label prescriptions (7.4% of total off-label prescriptions analysed). Nearly one third of individuals receiving off-label prescriptions of dexamethasone eye drops were under three years old, and over 50% were younger than five. Additionally, 30% of the population at risk for adverse events due to off-label prescriptions of budesonide nasal spray were also under five years old.

In case of budesonide 100 mcg nasal spray, the SmPC discloses another excipient, potassium sorbate (E 202) 500 mcg/dose, which acts as a preservative, but it is not listed as EMD by EMA^4^.

**Table 2:** Topical (non-cutaneous) off-label medicinal products including EMDs in their composition.

Medicinal product	Route of Administration	Number of prescriptions (% of total) *	ATC code	EMD	EMD quantity on SmPC
Dexamethasone eye drops	Eye	315,665 (6.4%)	S03C - Corticosteroids and antiinfectives in combination S03CA - Corticosteroids and anti-infectives in combination S03CA01 - Dexamethasone and antiinfectives	Benzalkonium chloride	0.04 mg/mL
Budesonide 100 mcg nasal spray	Nasal	49,162 (1%)	R01A - Decongestants and other nasal preparations for topical use R01AD - Corticosteroids R01AD05 - Budesonide	Polysorbate 80 (E 433)	Not disclosed

Notes: *Considering total off-label prescriptions analysed = 4,922,984. Calculation of % of total = [Number of prescriptions by medicinal product/Total number of off-label prescriptions] *100; Degree of harm of EMDs for pediatric individuals split into 3 categories, being “low” (white rows), “moderate” (light grey rows), and ”severe” (dark grey rows) | Abbreviations: ATC: Anatomical Therapeutic Chemical Classification System; EMD: Excipient of mandatory declaration; mcg: Microgram; mL: Millilitre; mg: Milligram; SmPC: Summary of Product Characteristics | Sources^6,8,11,12^:.

### Inhaled medicinal products

This category was represented by three medicinal products, accounting for a total of 326,796 off-label prescriptions (6.6% of total off-label prescriptions analysed) (Table 3). Almost 40% of the prescriptions analysed for inhaled budesonide were for individuals under five years old. In contrast, all prescriptions for the combination of formoterol and budesonide were issued for children aged 11.

**Table 3:** Inhaled off-label medicinal products including EMDs in their composition.

Medicinal product	Number of prescriptions (% of total) *	ATC code	EMD	EMD quantity on SmPC
Budesonide 100 mcg inhalator	78,435 (1.6%)	R01A - Decongestants and other nasal preparations for topical use R01AD - Corticosteroids R01AD05 - Budesonide	Ethanol	0.5 mg/inhalation
Budesonide 200 mcg inhalator	246,942 (5%)		Ethanol	0.5 mg/inhalation
Formoterol and budesonide 160 mcg/4.5 mcg inhalator	1,419 (< 1%)		Lactose	730 mcg/inhalation

Notes: *Considering total off-label prescriptions analysed = 4,922,984. Calculation of % of total = [Number of prescriptions by medicinal product/Total number of off-label prescriptions] *100; Degree of harm of EMDs for pediatric individuals split into 3 categories, being “low” (white rows), “moderate” (light grey rows), and ”severe” (dark grey rows) | Abbreviations: ATC: Anatomical Therapeutic Chemical Classification System; EMD: Excipient of mandatory declaration; mcg: Microgram; mg: Milligram; SmPC: Summary of Product Characteristics | Sources^6,8,11,12^:.

## Discussion

### General results

This is a novel study, providing evidence about unintentional EMDs intake from medicinal products that have been prescribed off-label in the under-18 population, a relevant safety concern that remains unaddressed, and the evidence in the literature about similar population-based studies is null to date. One of the most harmful EMDs to consider in the present study was ethanol, a commonly used solvent in oral liquid formulations for adults. Although it was not identified in any off-label prescriptions for oral medicinal products, it was present in inhaled and topical medicinal products, which may cause local effects, systemic effects, or both. In children, ethanol poses a significant risk of acute poisoning from accidental overdose and chronic toxicity with long-term use^4^.

It is noteworthy to highlight that almost 65% of off-label prescriptions analysed were concentrated in only nine medicinal products, which in turn did not contain in their formulation any EMD, which reduced somehow the safety burden on the population analysed. However, there were 1,736,684 off-label prescriptions that were needed from further analysis on potential harmful EMDs for the under-18 population.

There is evidence about off-label prescription in pediatrics^7^, official regulation on EMDs from regulatory authorities^4,13^, reviews on the use of excipients for pediatric medicinal products from a technological standpoint^11^, the World Health Organization (WHO) strong commitment with prevention of adverse events in children on the latest Global Safety Report in 2024^14^, and innovative initiatives like the “Safety and Toxicity of Excipients for Pediatrics” (STEP) database, which compiles the safety and toxicity information of excipients from selected information sources (e.g., clinical and non-clinical)^12^. However, there is still a need to harmonize this evidence and initiatives and raise awareness among healthcare professionals about the additional safety concerns that could be associated with off-label prescriptions in this vulnerable population. This should consider not only the dosing and pharmaceutical form, but also factors such as their composition.

A recent review carried out in Italy about a regional pediatric drug safety surveillance program deployed for 10 years revealed under-reporting of adverse events in case of off-label prescriptions^15^. The authors provided proofs for such inconsistent result, matching with other European countries experience, and which is mostly justified by the potential legal implications derived from off-label prescribing practices, especially in the younger vulnerable population.

In light of the findings from the present study, we recommend emphasizing the risks associated with the off-label use of medicinal products in the pediatric population. Furthermore, after thorough evaluation of off-label prescriptions, it would be advisable to incorporate excipient-related risk warnings into electronic prescribing systems and to systematically collect data on adverse events potentially linked to excipients. Importantly, these risks may extend beyond off-label use alone.

### Oral medicinal products

According to the EMDs analysed, the threshold established by EMA on all the excipients is zero, except for sodium (e.g., less than 1 mmol (23 mg) per dose; maximum daily dose of 2 g/day in 2–15 years old), sorbitol (maximum daily dose of 5 mg/kg in children *aged up to two* years old; 140 mg/kg in children over two years of age) and sucrose (from zero to five grams))^4,11^.

In case of oral formulations, the higher the content on active ingredient, the higher the risk of high doses of EMDs or potentially harmful excipients in general. This was clear in case of oral paracetamol, where 500 mg oral effervescent tablets contained 412 mg sodium/tablet whereas 1 g of the same pharmaceutical form increased to 567 mg the sodium content per tablet. In case of either orally disintegrating tablets or effervescent tablets of paracetamol, EMDs composition was similar (i.e., sodium benzoate plus sorbitol plus sodium as general rule). Considering that 23 mg of sodium per dose would be equal to absence of sodium, one of these formulations exceeded up to 25 times this threshold (e.g., 567 mg of sodium/tablet). Sodium is a clear example of excipients used in adult formulations which are not suitable at all for the youngest pediatric patients, bringing potential adverse events. Infants – particularly premature and low birth weight infants - are especially at risk due to their immature hepatic and renal systems for metabolism and excretion of sodium^11^. For instance, it is widely recognized and recommended that adding salt to meals for children up to two years old should be avoided; similarly, this principle should apply to medicinal products intake^16^. Most of the formulations exceeded the sodium safety threshold, for instance 330 mg/tablet (effervescent tablets of paracetamol 330 mg plus vitamin C), 412 mg/tablet (e.g., orally disintegrating tablets and effervescent tablets of paracetamol 500 mg), 567 mg/tablet (effervescent tablets of paracetamol 1 g) and 157.9 mg/tablet (acetylcysteine 600 mg effervescent tablets), which was almost seven times above the recommended threshold recommended by EMA^4^. On top of individual composition by tablet, those formulations exceeded the recommendations by far if taken two or three times a day, which is the usual posology in paracetamol when indicated for pain and fever episodes.

Sorbitol is a source of fructose, so it should be avoided in case of intolerance. Particularly babies and young children (under two years old) may not yet be diagnosed with this condition, so medicinal products containing this EMD should be avoided^4^. Nevertheless, it was identified use of oral solution and powder in newborns and children two years old in the present study. The content of sorbitol in the medicinal products analysed was not negligible, there were oral tablets ranging from 252 mg to 565.25 mg, and acetylcysteine powder with 675 mg/sachet. Oral acetylcysteine solution (40 mg/mL) also posed a high risk for patients.

In case of glucose, the quantity of the medicinal products analysed was quite variable but not negligible. From 41.5 mg per sachet to 323.20 mg/chewable tablet. Especially for children, glucose and sucrose may be harmful to the teeth, and its content should also be considered in the case of patients with diabetes mellitus^4^. In some formulations the content of sucrose was extremely high (560.10 mg/tablet in simethicone chewable tablets and 450 mg/mL in simethicone solution); in the latter, 90% of off-label prescriptions were issued for infants under five years of age. On top of diabetes and dental caries, sucrose should also be avoided in patients with rare hereditary conditions of fructose intolerance, glucose-galactose malabsorption or sucrase-isomaltase insufficiency^4^.

Similar situation comes up with lactose, which was identified from 3.1 mg per sachet to 67 mg/chewable tablet. Avoiding lactose is remarkable in case of intolerance to some sugars and with rare hereditary conditions of galactose intolerance, total lactase deficiency or glucose-galactose malabsorption^4^.

Despite the EMA’s efforts to harmonize the definition of EMDs and establish daily safety intake thresholds, inconsistencies remain. For example, artificial sweeteners like aspartame and saccharin are prohibited by the EU in foods for children aged under three years old, but they are present in several medicinal products authorized for these age groups. A similar situation applies to colouring agents; while their use in foods for children under three is prohibited, this restriction does not extend to medicinal products to date^17^. In general, for infants who have not started food diversification beyond breastfeeding, the intake of certain EMDs at early stages may trigger severe adverse events, allergies and intolerances that were unnoticed to date.

The medicinal products analysed contained between five to 20 mg of aspartame per tablet and 25 mg per sachet (powder). In case of calcium + vitamin D chewable tablets, 66% of off-label prescriptions were for infants under one year old, despite the lack of non-clinical nor clinical data available to assess aspartame use in infants under 12 weeks of age^4^. Also, the fact of being chewable tablets raised concerns the risk of choking, as these patients are still unable to chew.

Allergic reactions (possibly delayed) are quite common in the case of azo colouring agents and parahydroxybenzoates. Similarly with sulphites (e.g., lemon fragrance in acetylcysteine effervescent tablets), which should be avoided in asthmatic patients, since it could bring hypersensitivity reactions and bronchospasm^4^.

On a different note, oral liquid formulations, such as solutions and suspensions, are often given to young children because they are easy to swallow. However, many liquid formulations, like the acetylcysteine disclosed above, have not been adequately studied and approved for use in children, as disclosed on the SmPC.

Benzoic acid and benzoates may cause neonatal jaundice which may develop into kernicterus (e.g., non-conjugated bilirubin deposits in the brain tissue). The medicinal products analysed contained from 50 to 120 mg/tablet^4^.

Propylene glycol and its esters were present in acetylcysteine oral solution (strawberry/grenadine fragrance 11.2 mg/mL) and simethicone oral solution (25 mg/mL). It is recommended to avoid its use in children under four years of age, but in the present study it was extensively identified in children below two years of age. It is relevant to note that accumulation of propylene glycol can occur in neonates and young children as they cannot adequately metabolize and excrete the excipient^4,11^.

In summary, oral chewable tablets containing calcium and vitamin D (*n* = 4,308 off-label prescriptions; preventive treatment for bone loss, ATC A12AX), followed by acetylcysteine powder (*n* = 44,936 off-label prescriptions; mucolytic agent, ATC R05CB), account for the highest number of EMDs classified as posing a risk of severe harm (e.g., sorbitol, aspartame, lactose, sucrose, sodium)^10^.

### Topical medicinal products

Ethanol (227.8 mg/mL in the medicinal product analysed) may cause burning sensation on damaged skin. In neonates (pre-term and term newborn infants), high concentrations of ethanol may cause severe local reactions and systemic toxicity due to significant absorption through immature skin (especially under occlusion)^4^.

Many EMDs used in topical formulations have been classified with mid to low degree of harmfulness. Examples like benzalkonium chloride, benzyl alcohol, butylated hydroxytoluene, cetostearyl alcohol/cetyl alcohol, and stearyl alcohol may cause local skin reactions (e.g., contact dermatitis), irritation to the eyes and membranes, and increased sensitivity to natural and artificial sunlight.

Sodium lauryl sulphate is not usually disclosed in preparations, although it is normally 70%. It may cause local skin reactions (such as stinging or burning sensation) or increase skin reactions caused by other products when applied in the same area. The thickness of the skin varies considerably according to the body site and with age and can be an important factor in the sensitivity to sodium lauryl sulphate. Sensitivity will also vary according to the type of formulation (and effects of other excipients), concentration, contact time and patient population (children, hydration level, skin colour and disease like atopic dermatitis).

Fragrances that may contain allergens are not quantified on the medicinal products analysed (e.g., aurantiol plus limonene and fragrance rifskin 54193), both used in antifungal preparations. In addition to allergic reactions in sensitized patients, non-sensitized patients may become sensitized.

Terbinafine solution (*n* = 1,618 off-label prescriptions; antifungal, ATC D01A) is the medicinal product in this section with the highest potential risk of harm, due to the presence of ethanol in its composition^10^.

### Inhaled medicinal products

Ethanol 0.5 mg per dose of budesonide was likely to affect the study population, and 37% of the off-label prescriptions were issued for patients under five years. In general, the risk of ethanol in population under-18 is very high, due to neurotoxicity and the potential risk of accumulation, inducing adverse events, especially in younger children with low or immature metabolic capacity, who may feel sleepy, change their behaviour, and hinder their ability to concentrate and take part in physical activities^4^.

In case of lactose, despite the content being lower than 1 mg/dose in the combination of formoterol and budesonide, same considerations for use than for oral medicinal products should apply^4^.

All inhaled medicinal products within the decongestant and corticosteroid class (ATC R01AD) pose a safety risk for the pediatric population. For budesonide inhalers (*n* = 325,377 off-label prescriptions), this risk is associated with the presence of ethanol in their formulation, while for formoterol combined with budesonide, the concern arises from the inclusion of lactose^10^.

### Limitations

This study has some limitations that may result in an underestimation of off-label use, as it only considered prescriptions issued by pediatricians in primary health care. Prescriptions from other medical specialties and those from secondary care (such as hospitals) were not included. The study focused solely on the off-label use of authorized medicinal products for adults and did not include medical devices, compounded formulas, cosmetics, food supplements and medicinal plants. In addition, non-prescription medicinal products, including over-the-counter (self-care) medicinal product, were not accounted for. Lastly, the results indicate potential off-label use and additional safety burden from EMDs based on prescriptions, as dispensation data was not available.

To ensure accuracy in the analysis, only branded medicinal products were included, as prescriptions for unbranded medicinal products are issued by active ingredient and do not allow for identification of specific products. While this approach strengthens data reliability, it may lead to a conservative estimate of the overall impact of EMDs in the off-label use of medicinal products in the population under 18, suggesting that the true extent could be even greater.

The unavailability of dosage pattern information from medical records precludes the possibility of establishing a correlation between prescription data and EMD exposure.

To enhance the generalizability of the findings to other countries, it would be important to identify the most appropriate equivalents in terms of brand names, manufacturers and composition to ensure comparability.

## Conclusions

The findings of this study highlight the importance of addressing the risks associated with the off-label use of medicinal products in the pediatric population. In addition to careful evaluation of off-label prescribing, it is crucial to integrate excipient-related risk warnings into electronic prescribing systems and to implement routine monitoring and data collection on adverse events linked to excipients. Notably, the potential risks posed by excipients may extend beyond off-label scenarios, underscoring the need for broader pharmacovigilance efforts in pediatric care.

It is essential to note that neonates and infants up to five years old accounted for nearly 50% of all off-label prescriptions among the under-18 population, exposing them to additional safety risks from potentially harmful EMDs in these formulations. The vulnerability of these age groups, combined with the findings of this project, underscores the need for greater awareness and advocacy for the development of safer medicinal products for children. EMDs like ethanol, sodium, and sweeteners, either artificial or not, may lead to severe adverse events, causing permanent or long-term harm. The development of robust national surveillance strategies aimed at protecting pediatric patient safety are crucial, following age-specific and product-specific approaches; and not only in secondary care (e.g., hospital) but also in primary care setting.

## Electronic supplementary material

Below is the link to the electronic supplementary material.

Supplementary Material 1

## Data Availability

Data is provided within the manuscript or supplementary information files.

## Abbreviations

AEMPS: The Spanish Agency of Medicines and Medical Devices
ATC: Anatomical Therapeutic Chemical Classification System
CGCOF: General Council of Official Colleges of Pharmacists
CIMA: Medicine Online Information Centre of AEMPS
EMA: European Medicines Agency
EMD: Excipient of Mandatory Declaration
EU: European Union
g: Gram
GP: General practitioner
IU: International Units
kg: Kilogram
mcg: Microgram
mg: Milligram
mL: Millilitre
NRLS: National Reporting and Learning System
PL: Package Leaflet
ppm: Parts Per Million
SmPC: Summary of Product Characteristics
STEP: Safety and Toxicity of Excipients for Pediatrics
UK: United Kingdom
WHO: World Health Organization
