# Supplementary material for: Assessment of mandatory declaration excipients in pediatric off label prescriptions in Spain
Source: Sci Rep. 2025 Jul 19;15:26293. doi: 10.1038/s41598-025-11647-x (PMC12276334; doi:10.1038/s41598-025-11647-x)
Supplement: Supplementary file 1 — Supplementary Material 1 [file 41598_2025_11647_MOESM1_ESM.docx]

**Supplementary Material 1**

**Table S1.** Detail on 22 EMDs identified and their purpose on formulations

| **EMD** | **Function** | **Routes of Administration** | **Number of prescriptions*** |
| --- | --- | --- | --- |
| Aspartame (E 951) | Sweetening agent | Oral (chewable tablets, effervescent tablets, powder) | 95,768 |
| Azo colouring agents Amaranth (E 123) | Colouring agent | Oral (solution) | 237,335 |
| Benzalkonium chloride | Antimicrobial preservative  Antiseptic  Disinfectant  Penetration enhancer  Solubilizing agent  Wetting agent  Cationic surfactant | Eye (drops) | 315,667 |
| Benzoic acid (E 210) and benzoates (e.g., sodium benzoate (E 211)) | Antimicrobial preservative  Lubricant | (E 211)  Oral (effervescent tablets, oral disintegrating tablets) | 168,504 |
| Benzyl alcohol | Antimicrobial preservative  Disinfectant  Solvent | Topical cutaneous (emulsion, cream, medicated shampoo) | 346,029 |
| Butylated hydroxytoluene  (E 321) | Antioxidant | Topical cutaneous (cream) | 191,186 |
| Cetostearyl alcohol including Cetyl alcohol | Emollient  Emulsifier  Thickener | Topical cutaneous (cream) | 289,868 |
| Ethanol | Antimicrobial preservative  Penetration enhancer  Solvent | Topical cutaneous (solution) Inhaled | 326,995 |
| Fragrances containing allergens | Fragrance | Topical cutaneous (medicated shampoo, gel) | 48,857 |
| Glucose | Sweetener  Binder  Osmotic agent | Oral (chewable tablets, effervescent tablets, powder) | 104,637 |
| Lactose | Direct compression excipient  Dry powder inhaler carrier  Lyophilization aid  Diluent | Oral Inhaled (capsules, powder, chewable tablets) | 52,071 |
| Macrogolglycerol ricinoleate (castor oil polyoxyl)  Macrogolglycerol hydroxystearate (castor oil polyoxyl hydrogenated) | Surfactant  Thickener  Emulsifier | Topical cutaneous (emulsion, gel) | 142,303 |
| Parahydroxybenzoates and their esters  (E 216)  (E 217)  (E 218)  (E 219) | Preservative | (E 216) (E 218) Oral  (solution) | 575,544 |
|  |  | (E 217) (E 219) Oral (suspension) |  |
| Polysorbates | Dispersing agent  Emulsifying agent  Nonionic surfactant  Solubilizing agent  Suspending agent  Wetting agent | (E 432) PS 20  Oral (solution) | 78,639 |
|  |  | (E 433) PS 80  Nasal (spray) |  |
| Propylene glycol (E 1520) and esters of propylene glycol | Fragrance  Antimicrobial preservative  Disinfectant  Humectant  Plasticizer  Solvent  Stabilizing agent | Oral (solution) | 383,513 |
|  |  | Topical cutaneous (solution, medicated shampoo) |  |
| Sodium | Diverse**** | Oral (orally disintegrating tablets, chewable tablets, effervescent tablets, powder, solution, suspension) | 693,547 |
| Sodium lauryl sulphate | Anionic surfactant  Emulsifying agent  Cleanser  Foaming agent  Solubilizing agent  Penetration enhancer | Topical cutaneous (gel, medicated shampoo) | 136,413 |
| Sorbitol (E 420) | Sweetening agent  Stabilizer  Diluent  Humectant  Plasticizer | Oral (orally disintegrating tablets, chewable tablets, effervescent tablets, powder, solution, suspension) | 268,185 |
| Soya oil  Hydrogenated soya oil | Emulsifier | Oral (chewable tablets) | 4,308 |
| Stearyl alcohol | Emulsion stabilizer  Fragrance  Surfactant/Emulsifying agent | Topical cutaneous (cream) | 98,681 |
| Sucrose | Sweetening agent  Coating agent  Cryoprotectant  Suspending agent  Binder/diluent  Viscosity increasing agent | Oral (chewable tablets, solution, capsules) | 256,228 |
| Sulphites including metabisulphites | Fragrance | Oral (effervescent tablets) | 46,524 |

Notes: *Total number of prescriptions will not add up to 4,922,984 because many excipients are included in same medicinal product; **Oral disintegrating tablets, effervescent tablets and chewable tablets; ***Chewable tablets; ****Sodium was included in excipients for oral preparations like saccharin sodium (artificial sweetener), anhydrous sodium carbonate (desiccant in effervescent tablets), sodium hydrogen carbonate (effervescence, buffer), docusate sodium (surfactant, emulsifier, solubilizing agent), disodium edetate (chelating agent), sodium hydroxide (pH increaser), carmellose sodium (rheology modifier, binder, dispersant, film former); E 211 is sodium benzoate; E 216 is propyl p-hydroxybenzoate; E 217 is sodium propyl p-hydroxybenzoate; E 218 is methyl p-hydroxybenzoate; E 219 is sodium methyl p-hydroxybenzoate; E 432 is polysorbate 20; E 433 is polysorbate 80; Degree of harm of EMDs for pediatric individuals split into 3 categories, being “low” (white rows), “moderate” (light grey rows), and ”severe” (dark grey rows) | Abbreviations: EMD: Excipient of Mandatory Declaration; PS: Polysorbates | Sources: [6, 11-12]

**Supplementary Material 2**

**Table S2.** Detail on 14 oral off-label medicinal products including EMDs in their composition

| **Medicinal product** | **Number of prescriptions (% of total) *** | **ATC code** | **EMD** | **EMD quantity on SmPC** |
| --- | --- | --- | --- | --- |
| Paracetamol 500 mg orally disintegrating tablets | 79,031  (1.6%) | N02B - Other analgesics and antipyretics  n02be - anilides  n02be01 - paracetamol | Sodium benzoate (E 211) | 61 mg/tablet |
|  |  |  | Sorbitol (E 420) | 300 mg/tablet |
|  |  |  | Sodium | 412 mg sodium/tablet |
| Paracetamol 500 mg effervescent tablets | 33,014  (<1%) |  | Sodium benzoate (E 211) | 61 mg/tablet |
|  |  |  | Sorbitol (E 420) | 300 mg/tablet |
|  |  |  | Sodium | 412 mg sodium/tablet |
| Paracetamol 1 g effervescent tablets | 34,146  (<1%) |  | Sodium benzoate (E 211) | 120 mg/tablet |
|  |  |  | Sorbitol (E 420) | 252 mg/tablet |
|  |  |  | Sodium | 567 mg sodium/tablet |
| Paracetamol + Vitamin C 330 mg/200 mg effervescent tablets | 22,313  (<1%) | N02B - Other analgesics and antipyretics  N02BE - Anilides  N02BE51 - Paracetamol, combinations excluding psycholeptics | Sodium benzoate (E 211) | 50 mg/tablet |
|  |  |  | Sorbitol (E 420) | 300 mg/tablet |
|  |  |  | Sodium | 330 mg/tablet |
| Domperidone 1 mg/mL suspension | 29,477  (<1%) | A03F - Propellants  A03FA - Propellant  A03FA03 - Domperidone | Polysorbate 20  (E 432) | Not disclosed |
|  |  |  | Sodium methyl p-hydroxybenzoate (E 219) | 0.9 mg/mL |
|  |  |  | Sodium propyl p-hydroxybenzoate (E 217) | 0.1 mg/mL |
|  |  |  | Sorbitol (E 420) | 0.32 mg/mL |
|  |  |  | Sodium | 23 mg (1 mmol) sodium/dose |
| Calcium + Vitamin D 400 IU/1,500 mg chewable tablets | 4,308  (<1%) | A12A - Calcium  A12AX - Calcium, combinations with, vitamin d and/or other drugs | Sorbitol (E 420) | 565.25 mg/tablet |
|  |  |  | Aspartame  (E 951) | 5 mg/tablet |
|  |  |  | Lactose | 67 mg/tablet |
|  |  |  | Sucrose | 1.52 mg/tablet |
|  |  |  | Partially hydrogenated soya oil | 0.30 mg/tablet |
|  |  |  | Sodium | Not disclosed |
| Acetylcysteine 200 mg/5 mL solution | 20,959  (<1%) | R05C - Expectorants, excluding combinations with cough suppressants  R05CB - Mucolytics  R05CB01 - Acetylcysteine | Sorbitol (E 420) | 120 mg/mL |
|  |  |  | Propyl p-hydroxybenzoate (E 216) | 0.2 mg/mL |
|  |  |  | Methyl p-hydroxybenzoate (E 218) | 1.8 mg/mL |
|  |  |  | Propylene glycol (E 1520) and esters of propylene glycol | 11.2 mg/mL |
|  |  |  | Sodium | 6.55 mg/mL |
| Acetylcysteine 200 mg powder | 44,936  (<1%) |  | Aspartame (E 951) | 25 mg/tablet |
|  |  |  | Sorbitol (E 420) | 675 mg/sachet |
|  |  |  | Lactose | 3.1 mg/sachet |
|  |  |  | Glucose | 41.5 mg/sachet |
| Acetylcysteine 600 mg effervescent tablets | 40,684  (<1%) |  | Aspartame (E 951) | 20 mg/tablet |
|  |  |  | Sulphites including metabisulphites | <10 ppm/tablet |
|  |  |  | Glucose | <60 mg/tablet |
|  |  |  | Sodium | 157.9 mg/tablet |
| Acetylcysteine Forte 600 mg effervescent tablets | 5,840  (<1%) |  | Aspartame (E 951) | 20 mg/tablet |
|  |  |  | Sulphites including metabisulphites | <10 ppm/tablet |
|  |  |  | Glucose | <60 mg/tablet |
|  |  |  | Sodium | 157.9 mg/tablet |
| Simethicone 40 mg chewable tablets (mint flavour) | 3,018  (<1%) | A03A - Agents against functional disorders of the stomach  A03AX - Other agents against functional ailments of the stomach  A03AX13 - Silicones | Sucrose | 560.,10 mg/tablet (330.20 mg dextrates) |
|  |  |  | Glucose | 323.30 mg/tablet (330.20 mg dextrates) |
| Simethicone 40 mg chewable tablets | 10,159  (<1%) |  | Sucrose | 282.46 mg/tablet (166.50 mg dextrates) |
|  |  |  | Glucose | 158.17 mg/tablet (166.50 mg dextrates) |
| Simethicone 100 mg/mL solution | 237,336  (<1%) |  | Propyl p-hydroxybenzoate (E 216) | 0.20 mg/mL |
|  |  |  | Methyl p-hydroxybenzoate (E 218) | 1.80 mg/mL |
|  |  |  | Propylene glycol (E 1520) and esters of propylene glycol | 25 mg/mL |
|  |  |  | Azo colouring agents | 0.057 mg/mL |
|  |  |  | Sucrose | 450 mg/mL |
| Budesonide 3 mg gastro-resistant capsules | 1,408  (<1%) | A07E - Intestinal anti-inflammatory agents  A07EA - Locally acting corticosteroids  A07EA06 - Budesonide | Sucrose | 240 mg/capsule |
|  |  |  | (hydrated) Lactose | 12 mg/capsule |

Notes: *Considering total off-label prescriptions analysed = 4,922,984. Calculation of % of total = [Number of prescriptions by medicinal product / Total number of off-label prescriptions] *100; Degree of harm of EMDs for pediatric individuals split into 3 categories, being “low” (white rows), “moderate” (light grey rows), and ”severe” (dark grey rows) | Abbreviations: ATC: Anatomical Therapeutic Chemical Classification System; EMD: Excipient of Mandatory Declaration; IU: International Units; mcg: Microgram; mg: Milligram; mL: Millilitre; ppm: Parts Per Million; SmPC: Summary of Product Characteristics | Sources: [6, 8, 11-12]

**Supplementary Material 3**

**Table S3.** Detail on 9 topical (cutaneous use) off-label medicinal products including EMDs in their composition

| **Medicinal product** | **Number of prescriptions (% of total) *** | **ATC code** | **EMD** | **EMD quantity on SmPC** |
| --- | --- | --- | --- | --- |
| Methylprednisolone aceponate 0.10% emulsion | 96,418  (2%) | D07A - Corticosteroids, monopharmaceuticals  D07AC - Potent corticosteroids (Group III)  D07AC14 - Methylprednisolone aceponate | Benzyl alcohol | Not disclosed |
|  |  |  | Macrogol methylglucose dioleate | Not disclosed |
|  |  |  | Glycerol (E 422) | 10 g/dose |
| Methylprednisolone aceponate 0.10% cream | 191,186  (3.9%) |  | Cetostearyl alcohol including Cetyl alcohol | 25 mg/g |
|  |  |  | Benzyl alcohol | 10 mg/g |
|  |  |  | Butylated hydroxytoluene (E 321) | 0.006 mg/g |
| Ketoconazole 2% cream | 81,928  (1.7%) | D01A - Antifungals for topical use  D01AC - Imidazole and triazole derivatives  D01AC08 - Ketoconazole | Stearyl alcohol | 75 mg/g |
|  |  |  | Cetostearyl alcohol including Cetyl alcohol | 20 mg/g |
|  |  |  | Propylene glycol (E 1520) and esters of propylene glycol | 200 mg/g |
| Ketoconazole 20 mg/g gel | 45,885  (<1%) |  | Fragrances containing allergens | Not disclosed |
|  |  |  | Sodium lauryl sulphate | Not disclosed |
|  |  |  | Macrogolglycerol ricinoleate (castor oil polyoxyl)  Macrogolglycerol hydroxystearate (castor oil polyoxyl hydrogenated) | Not disclosed |
| Terbinafine 1% cream | 16,753  (<1%) | D01A - Antifungals for topical use  D01AE - Other antifungal preparations for topical use  D01AE15 - Terbinafine | Stearyl alcohol | 40 mg/g |
|  |  |  | Cetostearyl alcohol including Cetyl alcohol | 40 mg/g |
|  |  |  | Benzyl alcohol | 10 mg/g |
| Terbinafine 1% solution | 1,618  (<1%) |  | Propylene glycol (E 1520) and esters of propylene glycol | 48.54 mg/mL |
|  |  |  | Ethanol | 227.8 mg/mL |
| Ciclopirox 1.50% medicated shampoo** | 1,486  (<1%) | D01A - Antifungals for topical use  D01AE - Other antifungal preparations for topical use  D01AE14 - Ciclopirox | Sodium lauryl sulphate | 70% |
|  |  |  | Fragrances containing allergens | Not disclosed |
| Ciclopirox 1.50% medicated shampoo** | 1,486  (<1%) |  | Sodium lauryl sulphate | 70% |
|  |  |  | Fragrances containing allergens | Not disclosed |
| Ciclopirox 1.50% medicated shampoo** | 41,672  (<1%) |  | Sodium lauryl sulphate | 70% |
|  |  |  | Benzyl alcohol | 9 mg |
|  |  |  | Propylene glycol (E 1520) and esters of propylene glycol | Not disclosed |

Notes: *Considering total off-label prescriptions analysed = 4,922,984. Calculation of % of total = [Number of prescriptions by medicinal product / Total number of off-label prescriptions] *100; **Makes reference to three different brands with different EMD composition but same indications; Degree of harm of EMDs for pediatric individuals split into 3 categories, being “low” (white rows), “moderate” (light grey rows), and ”severe” (dark grey rows) | Abbreviations: ATC: Anatomical Therapeutic Chemical Classification System; EMD: Excipient of Mandatory Declaration; g: Gram; mcg: Microgram; mg: Milligram; mL: Millilitre; SmPC: Summary of Product Characteristics | Sources: [6, 8, 11-12]
